# Examining marginal sequence similarities between bacterial type III secretion system components and *Trypanosoma cruzi* surface proteins: horizontal gene transfer or convergent evolution?

**DOI:** 10.3389/fgene.2013.00143

**Published:** 2013-08-16

**Authors:** Danielle C. F. Silva, Richard C. Silva, Renata C. Ferreira, Marcelo R. S. Briones

**Affiliations:** ^1^Departamento de Microbiologia, Imunologia e Parasitologia, Universidade Federal de São PauloSão Paulo, Brazil; ^2^Laboratório de Genômica Evolutiva e Biocomplexidade, Universidade Federal de São PauloSão Paulo, Brazil; ^3^Departamento de Medicina, Disciplina de Infectologia, Universidade Federal de São PauloSão Paulo, Brazil

**Keywords:** horizontal gene transfer (HGT), evolution, *Trypanosoma cruzi*, *Salmonella* spp., Type III secretion system (T3SS)

## Abstract

The cell invasion mechanism of *Trypanosoma cruzi* has similarities with some intracellular bacterial taxa especially regarding calcium mobilization. This mechanism is not observed in other trypanosomatids, suggesting that the molecules involved in this type of cell invasion were a product of (1) acquisition by horizontal gene transfer (HGT); (2) secondary loss in the other trypanosomatid lineages of the mechanism inherited since the bifurcation Bacteria-Neomura (1.9 billion to 900 million years ago); or (3) *de novo* evolution from non-homologous proteins via convergent evolution. Similar to *T. cruzi*, several bacterial genera require increased host cell cytosolic calcium for intracellular invasion. Among intracellular bacteria, the mechanism of host cell invasion of genus *Salmonella* is the most similar to *T. cruzi*. The invasion of *Salmonella* occurs by contact with the host's cell surface and is mediated by the type III secretion system (T3SS) that promotes the contact-dependent translocation of effector proteins directly into host's cell cytoplasm. Here we provide evidence of distant sequence similarities and structurally conserved domains between *T. cruzi* and *Salmonella* spp T3SS proteins. Exhaustive database searches were directed to a wide range of intracellular bacteria and trypanosomatids, exploring sequence patterns for comparison of structural similarities and Bayesian phylogenies. Based on our data we hypothesize that *T. cruzi* acquired genes for calcium mobilization mediated invasion by ancient HGT from ancestral *Salmonella* lineages.

## Introduction

The protist *Trypanosoma cruzi* is a heteroxenic parasite and the causative agent of Chagas disease which represents an important public health problem in Latin America (WHO, 2010). Differently from other mammal infecting trypanosomatids, only *T. cruzi* can actively invade non-phagocytic host cells (Shi et al., 2004; El-Sayed et al., 2005b; Sibley, 2011). The cellular invasion mechanism of *T. cruzi* is remarkably similar to invasion mechanisms found in intracellular bacterial genera such as *Shigella* and *Salmonella*, especially regarding cellular calcium mobilization. Because these mechanisms are not observed in other trypanosomatids (Docampo and Moreno, 1996; Burleigh and Woolsey, 2002; Shi et al., 2004; El-Sayed et al., 2005b; Sibley, 2011) three possible explanations for the origin of *T. cruzi* calcium-dependent invasion mechanism can be conjectured: (1) the acquisition by horizontal gene transfer (HGT), (2) secondary loss in non-*T. cruzi* trypanosomatids, or (3) parallel or convergent evolution from non-homologous *T. cruzi* surface proteins.

The “TriTryps” sequencing genome project revealed bacterial kinase genes such as ribulokinase and galactokinases in *T. cruzi* and *Leishmania major* genome (El-Sayed et al., 2005b), consistent with the idea that these kinases were probably acquired by HGT from bacteria to trypanosomatids. Also, the hypothesis of HGT was tested to explain the similarity between *T. cruzi* trans-sialidases and bacterial sialidases (Briones et al., 1995). As a matter of fact, Opperdoes and Mitchels propose that the acquisition of a large number of foreign genes from viruses and bacteria was necessary for the evolution of trypanosomatids (Opperdoes and Michels, 2007).

Similarly to *T. cruzi*, increased host cell cytosolic calcium is required for intracellular invasion of several bacterial genera. Among intracellular bacteria, the mechanism of host cell invasion of genus *Salmonella* shares the highest similarities with *T. cruzi* (Clerc et al., 1989; Burleigh and Andrews, 1995; Collazo and Galán, 1997; Dramsi and Cossart, 1998; Suárez and Rüssmann, 1998; Burleigh and Woolsey, 2002; Andrade and Andrews, 2004; TranVan Nhieu et al., 2004). The invasion of *Salmonella* occurs by contact with the host's cell surface and is mediated by the type III secretion system (T3SS) that promotes the contact-dependent translocation of effector proteins directly into host's cell cytoplasm (Dramsi and Cossart, 1998; Mirold et al., 2001; Cossart and Sansonetti, 2004; TranVan Nhieu et al., 2004).

Here we performed exhaustive database searches directed to a wide range of intracellular bacteria and trypanosomatids, exploring sequence patterns and predicted secondary structures for comparison to detect even distant or marginal similarities between sequences and structures of *T. cruzi* that could be even remotely conserved with bacterial T3SSs. These conserved structures could be indicative of HGT or an extreme case of convergent evolution very specific in the *T. cruzi* lineage and completely absent in other trypanosomatids.

## Methods

### Database mining

#### Searches for genes similar to T. cruzi involved in intracellular bacterial invasion

Nucleotide sequences of genes encoding proteins SipD, SopB, SopD, and SopE2, present in all strains of genus *Salmonella* (Mirold et al., 2001) obtained in GeneDB (http://www.genedb.org/Homepage in September/2009), were used as BLASTN queries (Cummings et al., 2002) in completed intracellular bacterial (facultative or obligate) genome (http://www.genedb.org/Homepage in September/2009). New searches were performed in *T. cruzi* CL-Brener genome database (http://www.genedb.org/Homepage/Tcruzi in October/2009) using the nucleotide sequences from 57 strains of 11 genera and 28 intracellular bacterial species (including *Salmonella typhi*) obtained in the former search (Data Sheet 1 in Supplemental Data).

#### Searches for T. cruzi proteins similar to T3SS effector proteins from different bacteria

**Figure 1:**
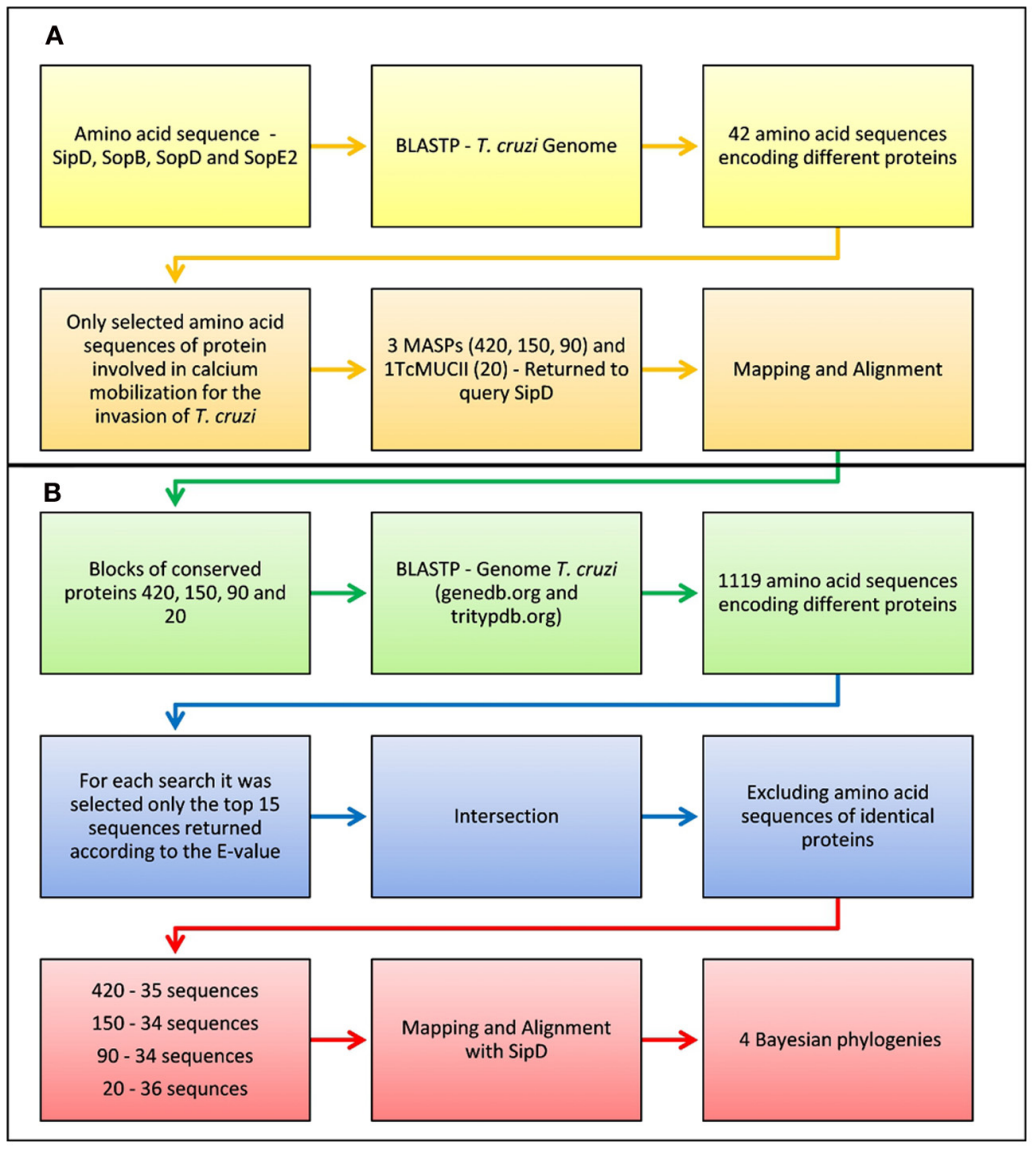
**Flowchart of the pipeline used in the analysis of sequence similarities between bacteria and trypanosomatids. (A)** Only the sequences of proteins whose role in calcium mobilization during *T. cruzi* invasion is currently known were selected. The amino acid sequences from T3SS proteins of *Escherichia coli* (EHEC O157:H7) str. EDL933, *Salmonella enterica* (serovar Typhi) str. CT18, *Shigella flexneri* (serotype 2a) str. 301, *Pseudomonas aeruginosa* PAO1, and *Yersinia pestis* CO92, downloaded from the Virulence Factors Database (http://www.mgc.ac.cn/VFs/ in March/2010) were also submitted to BLASTP (http://www.genedb.org/Homepage/Tcruzi in March/2010), being selected only the first 15 sequences according to their lower E-values. **(B)** The amino acid consensus sequences of *T. cruzi* proteins retrieved from BLASTP, TcCLB.508221.420, TcCLB.510693.150, TcCLB.511089.90, and TcCLB.506611.20 (designated as 420, 150, 90, and 20, respectively) were manually mapped and submitted again to BLASTP in the *T. cruzi* genome database GeneDB (http://www.genedb.org/Homepage/ in March/2010) and TriTrypDB—Esmeraldo-like and Non-Esmeraldo-like (http://tritrypdb.org/tritrypdb in April/2010), being selected only the first 15 non-redundant sequences according to their lower E-values.

Amino acid sequences of proteins SipD, SopB, SopD, and SopE2 were submitted to BLASTP (Cummings et al., 2002) in the *T. cruzi* CL-Brener protein database (http://www.genedb.org/Homepage/Tcruzi in September/2009). Only the sequences of proteins whose role in calcium mobilization during *T. cruzi* invasion is currently known were selected (Moreno et al., 1994; Acosta-Serrano et al., 2001; Villalta et al., 2008) (Figure 1A). The amino acid sequences from T3SS proteins of *Escherichia coli* (EHEC O157:H7) str. EDL933, *Salmonella enterica* (serovar Typhi) str. CT18, *Shigella flexneri* (serotype 2a) str. 301, *Pseudomonas aeruginosa* PAO1, and *Yersinia pestis* CO92, downloaded from the Virulence Factors Database (http://www.mgc.ac.cn/VFs/ in March/2010) were also submitted to BLASTP (http://www.genedb.org/Homepage/Tcruzi in March/2010), being selected only the first 15 sequences according to their lower E-values. The amino acid consensus sequences of *T. cruzi* proteins retrieved from BLASTP, TcCLB.508221.420, TcCLB.510693.150, TcCLB.511089.90, and TcCLB.506611.20 (from this point forward designated as 420, 150, 90, and 20, respectively) were manually mapped and submitted again to BLASTP in the *T. cruzi* genome database GeneDB (http://www.genedb.org/Homepage/ in March/2010) and TriTrypDB—Esmeraldo-like and Non-Esmeraldo-like (http://tritrypdb.org/tritrypdb in April/2010), being selected only the first 15 non-redundant sequences according to their lower E-values (Figure 1B).

#### Similarity searches in different protists

Amino acid sequence of *S. typhi* SipD was used as query in numerous searches with BLASTP in the genome database of *Bodo saltans*, *Trypanosoma brucei* gambiense, *T. brucei* 427, *T. brucei* 927, *Trypanosoma congolense*, *T. cruzi*, *Trypanosoma vivax*, *Leishmania mexicana, L. major* strain Friedlin, *Leishmania braziliensis* and *Leishmania infantum* in GeneDB and TritrypDB (www.genedb.org/Homepage/ and http://tritrypdb.org/tritrypdb in March/2011), *Euglena gracilis* (txid3039) and *Paramecium tetraurelia* strain d4-2 (txid412030) (http://blast.ncbi.nlm.nih.gov/Blast.cgi in June/2011). Only the first 15 non-redundant sequences were selected.

#### Similarities searches of trypanosomatids and S. typhi

Genome sequence of *S. typhi* CT18 (chromosome, plasmid 1 and 2) was downloaded from NCBI (http://www.ncbi.nlm.nih.gov/genomes/lproks.cgi in October/2011) and submitted to BLASTN algorithm in the *L. major* strain Friedlin, *T. brucei* strain 927 and *T. cruzi* strain CL Brener genome databases at GeneDB (http://www.genedb.org/Homepage in November/2011). Sequences encoding ubiquitous proteins such as heat shock and mitochondrial were discarded. Amino acid sequences of proteins SipD, SopB, SopD, and SopE2 of *S. typhi* were used as query in BLASTP searches in the genome database from *L. major* strain Friedlin and *T. brucei* strain 927 at GeneDB (http://www.genedb.org/Homepage in May/2012).

### Protein sequence alignments

The amino acid sequences were aligned using ClustalX (Thompson et al., 1997). For exclusive initial pairwise alignments were performed using default settings (matrix: Gonnet 250, gap opening = 10.00, and gap extension = 0.10). Multiple alignments were carried out with the following parameters: pairwise and multiple alignments using gap opening and gap extension = 1.00, being the alignment matrix modified to PAM 350 on the protists and trypanosomatids amino acid alignments. Multiple alignments of trypanosomatids and other protists were made using PAM350 matrix, which is most adequate for highly divergent sequences. This matrix is based on an explicit evolution model which takes into account the observed substitutions in a gobal alignment. Also, three different parameters were tested in multiple alignments: (1) pairwise gap opening (go) = 10.00 and gap extension (ge) = 0.10 and multiple go = 10.00 and ge = 0.20, (2)go = 1.00 and ge = 1.00, and (3) pairwise go = 35.00 and ge = 0.75 and multiple go = 15.00 and ge = 0.30. After evaluation of alignments with different parameters we chose go = 1.00 and ge = 1.00 because it maximized the number of conserved blocks. With other parameters the only blocks formed were between proteins in the same gene family where aminoacids are conserved. Also, parameters of type (3) above, yielded poor alignments with several blocks of unaligned sequences. This was used as preliminary approach and that is why it was not included in the manuscript. Therefore, Bayesian trees were not inferred using parameters as described in (1) and (3). For the loopback multiple alignments (420, 150, 90, and 20) the go and ge were both set to 1.00. The matrix was the Gonnet 250 because these were related sequences from the same organism in its majority from the same gene family (MASPs). Alignments were manually checked and adjusted using the Seaview4 sequence editor (Gouy et al., 2010).

### *In silico* analysis of deduced amino acid sequences

Secondary structure of proteins 420, 150, 90, 20, and SipD were analyzed using Geneious v5.5 (Drummond et al., 2011) with GOR1 method and idc = 3 (Garnier et al., 1978). Protein domain searches were performed in Pfam database (Finn et al., 2010). Sequences were also submitted to prediction servers at CBS (http://www.cbs.dtu.dk/services) for signal peptide (SP), transmembrane domains, function, and subcellular localization and Post-translational modifications such as N and O-glycosylation. Prediction of GPI-anchor sites (glycosylphosphatidylinositol) was performed by servers GPI-SOM (Fankhauser and Mäser, 2005) and PredGPI (Pierleoni et al., 2008). The membrane proteins were predicted using Mem Type-2L server (Chou and Shen, 2007). The presence of signal sequence of T3SS effector proteins was predicted at Modlab server (Löwer and Schneider, 2009).

### Codon usage and GC content analysis

Codon usage analysis was carried out with nucleotide sequences encoding for *S. typhi* SipD and *T. cruzi* proteins 420, 150, 90, 20, and actin (TcCLB.510573.10) using The Sequence Manipulation Suite (Stothard, 2000). The GC content was analyzed using the same sequences and also with their respective upstream and downstream intergenic regions using Geneious v5.5 (Drummond et al., 2011).

### Sequence variability

Sequence variability was measured using Shannon entropy (Shannon, 1948) with BioEdit v.7 program (Hall, 1999) for each position of the amino acid alignment from full sequences obtained in loopback searches and alignment with the conserved amino acid blocks used in Bayesian phylogenetic trees. Values obtained in nits were converted to bits by calculating the base 2 log of nit values.

### Phylogenetic inference

Phylogenetic trees were inferred from amino acid sequence alignments retrieved from BLASTP (Data Sheet 1 in Supplemental Data) and from alignments generated from database searches of different protists (*B. saltans*, *E. gracilis, L. mexicana*, *L. major*, *L. braziliensis* e *L. infantum*, *P. tetraurelia T. brucei gambiense*, *T. brucei 427, T. brucei 927*, *T. cruzi, T. congolense*, and *T. vivax)*, using MrBayes v3.1.2 (Huelsenbeck et al., 2001). MCMC algorithm started from a random tree, estimating the amino acids substitution model. Trees were inferred from 3 × 10^7^ generations sampling a tree in every 100 generation until the standard deviation from split frequencies were under 0.01. The parameters and the trees were summarized by wasting at least 25% of the samples obtained (burnin). The consensus trees were then used to determine the posterior probabilities values. All phylogenetic trees were then formatted with the FigTree v1.3.1 program (http://tree.bio.ed.ac.uk/software/figtree/).

## Results and discussion

### Proteins involved in intracellular invasion similar to *T. cruzi* proteins

Among all bacterial genera analyzed (Data Sheet 1 in Supplemental Data), positive BLASTN results were obtained only for genera *Bordetella*, *Chlamydophila*, and *Shigella*. These sequences, along with sequences encoding proteins SipD, SopB, SopD e SopE2 of *S. typhi* were used as queries for searches in the *T. cruzi* genome database. A total of 689 open reading frames (ORFs) were retrieved. Sequences whose *in silico* translation included frameshifts and/or unrelated amino acids, were excluded. Only amino acid sequences obtained by BLASTP were used for further analysis.

BLASTP searches were then performed using as queries the amino acid sequences of the *S. typhi* effector proteins SipD, SopB, SopD, and SopE2 against the *L. major*, *T. brucei*, and *T. cruzi* genome database, yielding 21, 24, and 42 sequences, respectively. From these sequences, we performed predictions to determine their possible locations and functions (Data Sheet 3 in Supplemental Data). We show that the number of *T. cruzi* amino acid sequences potentially involved in the invasion mechanism was superior to other trypanosomatids. Two sequences with the potential to be on the parasite surface were found both in *L. major* and in *T. brucei* (Data Sheet 3 in Supplemental Data). However, they were not analyzed further because they are classified as hypothetical or pseudogenes and because it is already known that both parasites do not mobilize intracellular calcium during invasion and thus cannot actively invade host cells (Shi et al., 2004; El-Sayed et al., 2005b; Sibley, 2011). Prediction analysis of *T. cruzi* BLASTP results output showed that 9 sequences had the potential to be involved in host cell invasion (Data Sheet 3 in Supplemental Data). Among those, only the putative sequences of mucins and/or mucin associated surface proteins (MASP) (420, 150, 90, and 20) were selected because of their already known involvement with calcium mobilization during *T*. *cruzi* cell invasion (Moreno et al., 1994; Acosta-Serrano et al., 2001; Villalta et al., 2008). We discarded search hits of proteins whose involvement in *T. cruzi* cell invasion has not yet been demonstrated to increase the chance to detect marginal similarities among proteins associated with this mechanism (Figure 1B). Positive database search results were only obtained with protein SipD. This protein is known to increase the level of proteins secreted by the T3SS and plays a crucial role in *Salmonella* host cell invasion. Its absence causes the complete impairment of effector proteins translocation and hinders the invasion process (Kubori and Galán, 2002). *T. cruzi* MASPs and mucins and bacterial SipD are expressed on cell surface even before invasion, although these can also be found in the cytosol and are intimately involved with mechanisms of pathogenicity (Acosta-Serrano et al., 2001; Kubori and Galán, 2002; Eswarappa et al., 2008; Villalta et al., 2008; De Pablos et al., 2011). These data suggest the homology among SipD, MASPs, and mucins, and also suggest that their functions in calcium mobilization might be conserved (Henikoff and Henikoff, 1992).

In an attempt to find proteins similar to MASPs and mucins in other T3SS bacteria and not restrict the analysis to proteins associated with calcium mobilization of genus *Salmonella*, we performed new searches against the *T. cruzi* genome database with amino acid sequences from different bacterial T3SS (Data Sheet 4 in Supplemental Data). These searches revealed a considerable number of MASPs and mucins (Table 1). Our results are consistent with the hypothesis of HGT of T3SS genes to *T. cruzi* because BLAST results of MASPs and mucins are not unique to *Salmonella* queries. However, because the percentage of MASPs returned by searches with *Salmonella* was significantly higher, sequences from other genera were not further analyzed (Table 1). Also, when comparing the invasion mechanisms associated with different T3SS, *Salmonella* shows the highest similarity with *T. cruzi*. Both organisms can invade non-phagocytic cells, use inositol 1,4,5-trisphosphate (IP3) to elevate intracellular calcium and consequently induce cytoskeleton rearrangement and remain inside vacuoles during the first stages of cell invasion (Clerc et al., 1989; Burleigh and Andrews, 1995; Collazo and Galán, 1997; Dramsi and Cossart, 1998; Suárez and Rüssmann, 1998; Burleigh and Woolsey, 2002; Andrade and Andrews, 2004; TranVan Nhieu et al., 2004). Although other bacteria share some of these mechanisms, genus *Salmonella* shares most of the observed features. The host cell invasion mechanism of *Shigella* is relatively similar to *Salmonella* (Dramsi and Cossart, 1998) and involves T3SS proteins (Espina et al., 2006; Parsot, 2009) but differs from *T. cruzi* because it does not exclusively depend on intracellular calcium mobilization and does not remain in vacuoles during the first stages of invasion (Clerc et al., 1989; Collazo and Galán, 1997).

**Table 1:** **Database searches using amino acid sequences of the T3SS proteins of different bacteria**.

**Bacteria**	**T3SS Proteins**	**MASP**	**TcMUCII**	**Others**	**MASP (%)**
*E. coli*	18	22	8	103	13.53
*S. typhi*	8	16	2	50	23.53
*S. flexneri*	6	11	3	61	14.66
*P. aeruginosa*	37	23	3	263	7.96
*Y. pestis*	41	20	10	332	5.52

To verify if the marginal sequence similarities between bacteria and *T. cruzi* are specific to genes encoding T3SS proteins, searches using the whole *S. typhi* genome as query were performed against the genome databases from different members of Trypanosomatidae (Table 2). These searches returned a large number of sequences coding for common proteins shared by all classes of eukaryotic organisms such as mitochondrial and heat shock proteins. These searches also returned several genes encoding hypothetical proteins and stage-specific proteins of each parasite (data not shown). However, these genes were not considered as positive hits for possible “trace-homologies” that could be involved with infectivity, because negative results were obtained when predictions for subcellular localization, SP, and GPI anchoring were performed with their deduced amino acid sequence (data not shown), suggesting that these putative proteins are possibly not secreted or present on the cell surface. These results are supported by the fact that *T. cruzi* adhesion and invasion does not seem to be simple i.e., involving a single ligand-receptor interaction. Trypomastigotes exploit a huge palette of surface glycoproteins, secreted proteases, and agonist signaling to actively manipulate the host cell invasion (Burleigh and Andrews, 1995; Di Noia et al., 1998; Acosta-Serrano et al., 2001; Burleigh and Woolsey, 2002; Buscaglia et al., 2006; Yoshida, 2006; Villalta et al., 2008). As expected, searches in the *T. cruzi* genome database using the whole *S. typhi* genome returned several sequences that encode proteins involved in host cell adhesion/invasion such as DGF-1 (Dispersed Gene Family 1) and MASPs (Moreno et al., 1994; Acosta-Serrano et al., 2001; Villalta et al., 2008; Kawashita et al., 2009) (Data Sheet 2 in Supplemental Data).

**Table 2:** **Comparative genome analysis of *S. typhi* and trypanosomatids**.

***S. typhi***	***T. cruzi***			***T. brucei***			***L. major***		
	**Surface**	**Hypothetical**	**Common**	**Surface**	**Hypothetical**	**Common**	**Surface**	**Hypothetical**	**Common**
Chromosome	9 (MASPs)	5	86	0	2	98	0	2	99
Plasmid 1	97 (DGF-1)	1	2	0	0	4	0	73	31
Plasmid 2	3 (MASPs)	0	1	0	1	1	0	2	4
Total	109	6	89	0	3	103	0	77	134

### Amino acid sequences similarities

**Figure 2:**
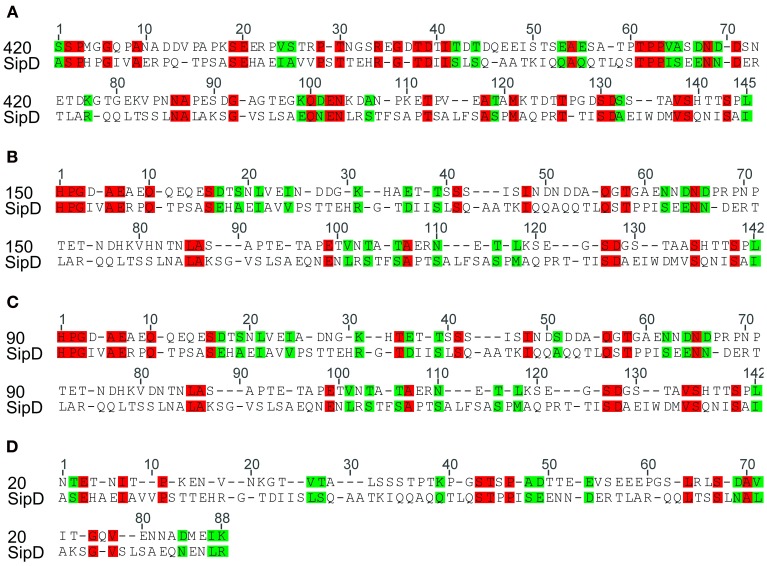
**Similarity between *Salmonella typhi Sip*D and *T. cruzi* proteins.** The identity and similarity between the aligned sequences. Red represents identical residues and green indicates conservative changes. Local amino acid sequences were initially aligned using ClustalX (Thompson et al., 1997). Pairwise alignments were performed with default settings (see Methods) and adjusted manually in Seaview sequence editor (Gouy et al., 2010). **(A)**, **(B)**, **(C),** and **(D)** refers to the local alignment of the amino acid sequence of the protein SipD with MASPs 420, 150, 90, and mucin 20, respectively.

The complete amino acid sequences of *S. typhi* SipD and of *T. cruzi* MASPs and mucins (420, 150, 90, and 20) were aligned. As expected, due to the high rate of divergence among sequences, it resulted in few conserved blocks and positions embedded in highly divergent domains (data not shown). However, the mapping of local amino acid residues (local alignment) resulted in an alignment with good quality (pairwise identity, identical sites and similarities above 13, 16, and 29%, respectively) (Table 3) showing potential homologous positions (Figure 2). Alignments often provide important insights into protein functional mechanisms being the pairwise alignment of blocks a better option to perform homology searches (Henikoff and Henikoff, 1992; Batzoglou, 2005). SipD has residues important for *Salmonella* invasion. Although most of functional residues are located at the C-terminal, the portion of N-terminal which aligns with the *T. cruzi* proteins also has important sites, both by decreasing the invasion itself and by involvement with bile salts that suppress the *Salmonella* invasion (Wang et al., 2010; Chatterjee et al., 2011). Although most of the transferred genes are non-functional in the recipient genome, Woolfit et al. (2009) suggest that independently of the direction of the HGT, transferred genes may remain functional. These propositions are supported by different authors that argue that these genes are really important in the adaptation to new niches, to originate novel functions and for virulence (Opperdoes and Michels, 2007; Keeling and Palmer, 2008; Andersson, 2009; Cohen et al., 2011).

**Table 3:** **Sequence similarities between *Salmonella* SipD and *T. cruzi* MASPs and mucin**.

**Alignment**	**Positions**	**Identical**	**Pairwise**	**Similarity (%)**
		**sites (%)**	**identity (%)**	
SipD × 420	145	24.8	23.8	37
SipD × 150	142	18.3	14.7	30
SipD × 90	142	19.7	16.2	32
SipD × 20	88	15.9	12.9	29

*Similarity percentages were calculated using Geneious v5.5. software*.

### *In silico* analysis of protein structure and motifs

To verify possible homologies (“trace-homologies”) between *T. cruzi* and *Salmonella* proteins and also address the possible structural and functional properties shared by them, amino acid sequences were analyzed by different prediction methods. Searches for known sequence motifs and domains from manually curated databases using the amino acid sequences of proteins 420, 150, 90, and 20 from *T. cruzi* and the sequence of *S. typhi* SipD, showed that no characterized domains or motifs are present (data not shown). However, our predictions showed that SipD is part of the IpaD family, effector proteins from *Shigella* that share similar functional roles with SipD (Espina et al., 2006; Parsot, 2009).

**Figure 3:**
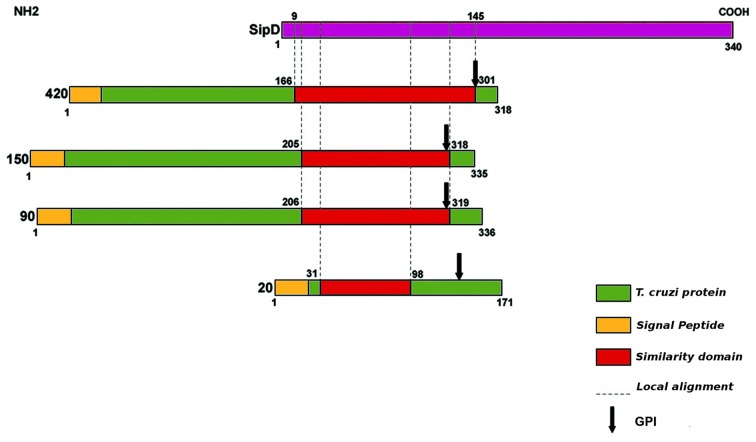
**Schematic illustration of amino acid sequence similarity between SipD (purple) and *T. cruzi* proteins (green).** Protein domain searches were performed in Pfam database (Finn et al., 2010). Sequences were also analysed at CBS (http://www.cbs.dtu.dk/services) for signal peptide (SP), transmembrane domains, function, and subcellular localization, and Post-translational modifications such as N and O-glycosylation. GPI-anchor sites (glycosylphosphatidylinositol) was predicted by GPI-SOM (Fankhauser and Mäser, 2005) and PredGPI (Pierleoni et al., 2008). The membrane proteins were predicted using Mem Type-2L server (Chou and Shen, 2007). The presence of signal sequence of T3SS effector proteins was predicted by Modlab (Löwer and Schneider, 2009).

As expected, SipD does not present a canonical SP because proteins from the T3SS are secreted through a sec-independent mechanism (Büttner and Bonas, 2002). The proteins 420, 150, 90, and 20 from *T. cruzi* present potential cleavage sites in positions 21 and 22, 25 and 26, 26 and 27, and 24 and 25, respectively. More importantly, the fact that the possible signal sequences in these proteins remain outside amino acid blocks that aligns with SipD (Figure 3) suggests that these residues are not cleaved during secretion. Predictions also suggest that proteins 420 and 90 possess possible transmembrane helices between positions 7 and 29, overlapping with their signal sequences. According to Bendtsen et al. (2004), transmembrane helices must be disregarded in these cases because signal sequences interfere with these predictions, leading to false positives. In addition, it is known that MASPs are GPI-anchored (Acosta-Serrano et al., 2001; Buscaglia et al., 2006) and that GPI-anchored proteins lack the transmembrane domains (Elortza et al., 2003).

We also found potential GPI anchoring sites in *T. cruzi* proteins 420, 150, 90, and 20 in positions 291, 305, 306, and 145, respectively. As a negative control, the amino acid sequence of SipD was used in this prediction. These data confirm our results because it is already known that MASPs and mucins are GPI-anchored proteins (Acosta-Serrano et al., 2001; Buscaglia et al., 2006). The potential GPI anchor sites of putative MASPs 420, 150, and 90 are localized at the end of the amino acid sequences that align with SipD. On the other hand, the predicted GPI-anchor site of putative mucin 20 differs from other proteins (Figure 3), suggesting a potential specialized and/or functional role of this specific site in these MASPs, and supporting their involvement with host-parasite interactions (Elortza et al., 2003; Epting et al., 2010).

In addition to the comparative results obtained with SipD, putative post translational modifications were analyzed (Table 4). Not surprisingly, the predictions are consistent with already known characteristics of this protein class (Acosta-Serrano et al., 2001; Buscaglia et al., 2006; Bartholomeu et al., 2009).

**Table 4:** **Predictions of protein sequence features**.

**Prediction**	**SipD**	**420**	**150**	**90**	**20**
Signal peptide	No	Yes	Yes	Yes	Yes
Transmembrane helix	No	Yes	No	Yes	No
GPI anchors	No	Yes	Yes	Yes	Yes
N-Glycosylation	No	2	3	3	2
O-Glycosylation	No	32	25	26	38

*The numbers indicate the sites predicted*.

**Figure 4:**
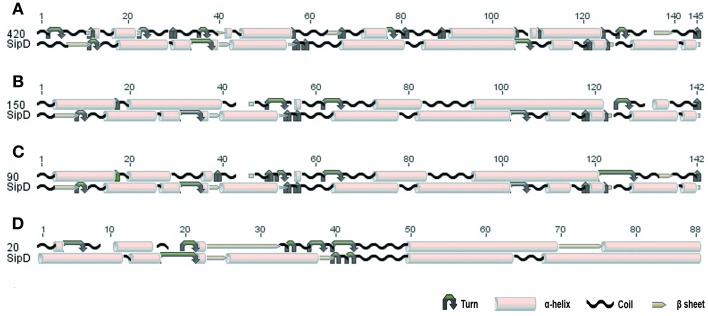
**Conserved secondary structure of the aligned blocks of proteins (A) 420, (B) 150, (C) 90, and (D) 20 of *T. cruzi*** with the SipD. Secondary structure of proteins 420, 150, 90, 20, and SipD were analyzed using the GOR1 method and idc = 3 (Garnier et al., 1978).

The comparison of protein structures is important to reveal evolutionary relationships among proteins. Protein families tend to be structurally conserved and these structures may be maintained even when sequences have diverged beyond any recognizable similarity (Orengo et al., 1997; Wieser and Niranjan, 2009; Joseph et al., 2011). To verify if the putative *T. cruzi* proteins and *S. typhi* SipD possess conserved secondary structural domains, their local amino acid sequences were analyzed. These local conserved residues are, in general, rare in regions containing sequences of amino acids forming beta-sheets and rich in alpha-helices and coil structures (Figure 4). The secondary structure of SipD maintains a similarity of approximately 30–45% with *T. cruzi* proteins (Table 5). Considering the phylogenetic distance between these organisms, it is reasonable to propose that these levels of secondary structure similarities might indicate homology. However, the quantification of secondary structure predictions should be taken carefully because the current software works with a confidence level of approximately 70% (Garnier et al., 1978; Creighton, 1990; Joseph et al., 2011). Nevertheless, our data indicate that the secondary structures of the conserved amino acid regions of *T. cruzi* and *S. typhi* are more conserved than the primary structure (Table 1), mostly because the secondary structure can be maintained even in regions where amino acids are not identical, *via* conservative amino acid substitutions.

**Table 5:** **Comparison of primary and secondary structure similarities**.

**Sequences**	**Primary structure**			**Secondary structure**					**Similarity (%)**	
	**Conserved**	**Identical**	**Similar**	**Conserved**	**α-helix**	**β sheet**	**Coil**	**Turn**	**Primary**	**Secondary**
SipD X 420	52	34	18	61	40	3	14	4	37.96	44.53
SipD X 150	37	20	17	47	38	0	9	0	27.01	34.31
SipD X 90	40	22	18	43	33	0	9	1	29.20	31.39
SipD X 20	23	11	12	48	35	3	7	3	16.79	35.79

*Data were generated from 137 positions respective to SipD*.

### Horizontal gene transfer and invasion mechanisms

Although HGT is recognized as an important evolutionary mechanism, its impact has been neglected and confused with mere phylogenetic noise in favor of a vertical signal resulting from the transmission of information from ancestors to descendants (Comas et al., 2006).

In view of the amino acid similarities and function, shared by *S. typhi* and *T. cruzi* proteins here presented and because this parasite is the only trypanosomatid that can actively invade host cells (Docampo and Moreno, 1996; Burleigh and Woolsey, 2002; Shi et al., 2004; El-Sayed et al., 2005b; Sibley, 2011), we propose the hypothesis of ancient HGT for the origin of calcium-dependent invasion mechanism of *T. cruzi*. It can be speculated that these ancient HGT events might have occurred by: (1) the ingestion of blood contaminated with *Salmonella* spp. or some other T3SS intracellular bacteria by species of *Triatominae* and the insertion of bacterial genes into the *T. cruzi* genome or (2) insertions and/or gene exchange by endosymbiotic bacteria. We also do not exclude that other trypanosomatids lost their ability to invade since the Bacteria-Neomura bifurcation (secondary loss). Nevertheless, the occurrence of multiple HGT events from bacterial endosymbionts in plants to trypanosomatids described by Hannaert et al. (2003) and by the possible occurrence of HGT in trypanosomatids originated from bacteria present in the intestine of *Triatominae* (Opperdoes and Michels, 2007).

**Figure 5:**
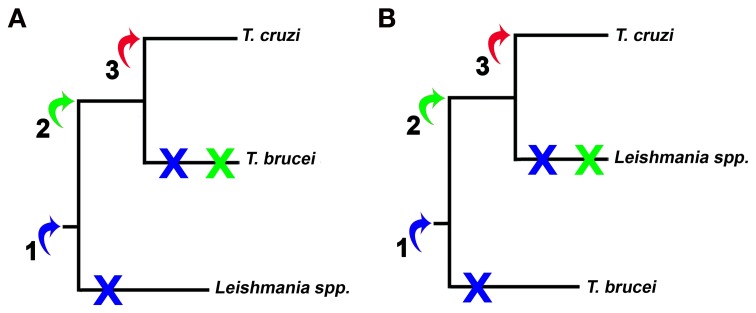
**Representation of HGT hypothesized in this work.** The arrows and numbers represent the possible insertion of bacterial genes. **(A)** and **(B)** represent the branching order of the trees of trypanosomatids considered in this work. Early HGT implies one acquisition (blue arrow) and two losses (blue X), while HGT in the *Trypanosoma* genus would imply one acquisition and one loss (green arrow and X). The most parsimonious hypothesis, or late HGT in *T. cruzi*, implies only one acquisition and no character loss (red arrow).

Here we examine three possibilities of HGT, summarized two different scenarios, monophyletic (Figure 5A) and paraphyletic (Figure 5B). Although most studies agree with the monophyly of the trypanosomatids, this issue remains controversial (Simpson et al., 2006; Leonard et al., 2011). Firstly, we supposed that this event might have occurred at point 1, being the genes transferred from one ancestor to all trypanosomatids. Therefore, all trypanosomatids would carry genes involved in calcium-dependent host cell invasion, but during evolution these genes could have been lost or silenced. Secondly, if HGT occurred at the point 2, genes would be present only in *T. cruzi* and *T. brucei* spp. (Figure 5A) or if we consider the trypanosomatids family tree presented in Figure 5B, genes would be present only in *T. cruzi* and *Leishmania* spp. Finally, if the transfer occurred at the point 3, only *T. cruzi* would have acquired the genes to actively invade host cell. Among these three hypotheses, we believe that the third has the highest likelihood due to the relative similarity of the host cell invasion mechanisms of bacteria, such as *Salmonella*, and *T. cruzi* (Clerc et al., 1989; Burleigh and Andrews, 1995; Collazo and Galán, 1997; Dramsi and Cossart, 1998; Suárez and Rüssmann, 1998; Burleigh and Woolsey, 2002; Andrade and Andrews, 2004; TranVan Nhieu et al., 2004) and absence of even remotely similar sequences in *T. brucei* and *Leishmania*. In addition, this is the most parsimonious hypothesis because it involves only one acquisition whereas the other hypotheses involve one acquisition and at least one secondary loss (Figure 4). This hypothesis is also supported by computational predictions (Data Sheet 3 in Supplemental Data), by the highly superior number of sequences obtained in database searches within *T. cruzi* genome database and by the potential of these sequences to be involved in invasion mechanisms. Although in small numbers, searches against the genome of *L. major* and *T. brucei* also returned 2 amino acid sequences. This may suggest that HGT occurred in a trypanosomatid common ancestor and that other trypanosomatids have lost this mechanism. The vertical inheritance would imply a loss dating to the bifurcation Bacteria-Neomura between 1.9 billion and 900 million years ago (Proterozoic Eon) (Cavalier-Smith, 1998).

There are different ways to detect patterns and signs of HGT events. In general they are based on bio-computational analysis, including homology searches, codon usage, and GC content analysis and phylogenetic inference (Cohen and Pupko, 2010; Li et al., 2011). Most commonly these approaches search for the distribution of atypical genes in different organisms and may include the identification of: (a) genes with highly restricted distributions, present in isolated *taxa* but absent from closely related species, (b) highly similar genes, and (c) genes whose phylogenies are incongruent with the relationships inferred from other genes in their respective genomes (Gogarten et al., 2002). Nonetheless, most methods used to evidence HGT are based on recent events, since ancient HGT events are harder to detect and genes may lose ancestor signatures through evolution. Phylogenetic inference of a broad range of sequences, though, may reveal ancient HGTs (McDonald et al., 2012), being considered as gold-standards.

**Figure 6:**
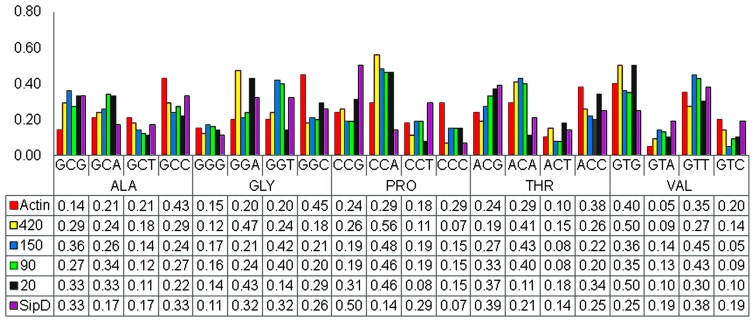
**Codon usage profiles.** The pattern of codon usage was obtained from the nucleotide sequences coding for proteins SipD, 420, 150, 90, 20, and the actin gene within The Sequence Manipulation Suite (Stothard, 2000). The charts were plotted with the Excel program. The abscissa indicates the four-fold degenerated amino acids and the ordinate represents the codon frequencies. Bars represent each codon used by the respective gene, and the values below the chart indicate the frequency of each codon in the respective genes.

Parametric analysis such as codon usage and GC content profiles are preferentially used to detect recent HGT events (Becq et al., 2010). We analyzed the codon usage profiles of nucleotide sequences encoding the putative *T. cruzi* proteins and *Salmonella* SipD. These analyses were performed with the four-fold degenerated amino acids only. These results did not strongly indicate the occurrence of HGT, but it is noticeable that the codon usage pattern of actin differ from other *T. cruzi* genes (Figure 6), suggesting a possible HGT event. Although SipD has a different codon usage profile in comparison to *T. cruzi* genes, this cannot be considered a negative result, since highly divergent genes tend to lose features from their ancestors (Philippe and Douady, 2003; McDonald et al., 2012). Additionally, transferred genes tend to behave homogeneously, similar to genes from the receptor organism. Thus, codon usage analyses are not sensitive enough to distinguish ancient HGT (Koski et al., 2001; Philippe and Douady, 2003). Therefore, if we look carefully it is possible to note that the frequencies of G and C levels in third codon positions are relatively close among genes encoding the *T. cruzi* proteins 420, 150, 90, and 20 and *S. typhi* SipD, in comparison to values of *T. cruzi* actin gene, mainly for the amino acids alanine (ALA), proline (PRO), and threonine (THR) (Figure 6). Usually vertically inherited genes are adapted to the codon usage characteristic of their original genome and expression level. On the other hand, horizontally acquired genes frequently have atypical G and C base compositions (Karberg et al., 2011). Together these results support the hypothesis that these *T. cruzi* genes were acquired by HGT, because they have different sequence features when compared to the actin gene.

Gene fixation in the HGT receptor organism requires a progressive compatibility of GC content and codon usage (Medrano-Soto et al., 2004). This criterion is used in the analysis of *T. cruzi* and *S. typhi* genes in this study, both with approximately 51% GC content (Parkhill et al., 2001; El-Sayed et al., 2005a). However, most methods identify horizontally transferred genes based on the identification of atypical GC content in DNA sequences (Becq et al., 2010; Karberg et al., 2011). The presence of atypical GC content in intergenic regions may reveal horizontally transferred genome islands (Kurup et al., 2010). Our results demonstrated that some values were in proximity to the GC content of intergenic and coding regions of each gene, except for the intergenic regions of actin (Table 6). It is known that MASPs and mucins, as well as some other surface proteins, unique to *T. cruzi*, are encoded by non-sintenic islands (El-Sayed et al., 2005b). Although we have not observed atypical GC content in intergenic regions between the possible genes acquired by horizontal transfer, we do not consider this as a negative result for a possible HGT event, particularly because methods to identify atypical sequences are limited to detection of recent transfers (Gogarten et al., 2002) and also because intergenic regions showed lower GC content than the other regions (Table 6). Gene content varies along a genome, and the number of members in each gene family. The difference in gene repertoire between the genomes of the same family and/or species is generally attributed to gene loss or HGT (Daubin and Ochman, 2004). Thus, we can assume that *T. cruzi* may have acquired a large number of foreign genes, since the size of its genome is approximately 20 Mb greater than the genomes of *T. brucei* and *L. major*, and MASPs and mucins are encoded within large genomic islands (El-Sayed et al., 2005b).

**Table 6:** **GC content of *T. cruzi* genes and intergenic regions (IG)**.

**Gene**	**GC content (%)**		
	**Coding**	**IG upstream**	**IG downstream**
420	50.7	52.2	52.5
150	52.0	50.8	58.2
90	51.6	52.6	54.6
20	55.4	55.3	48.2
Actin	51.7	32.0	36.1

**Figure 7:**
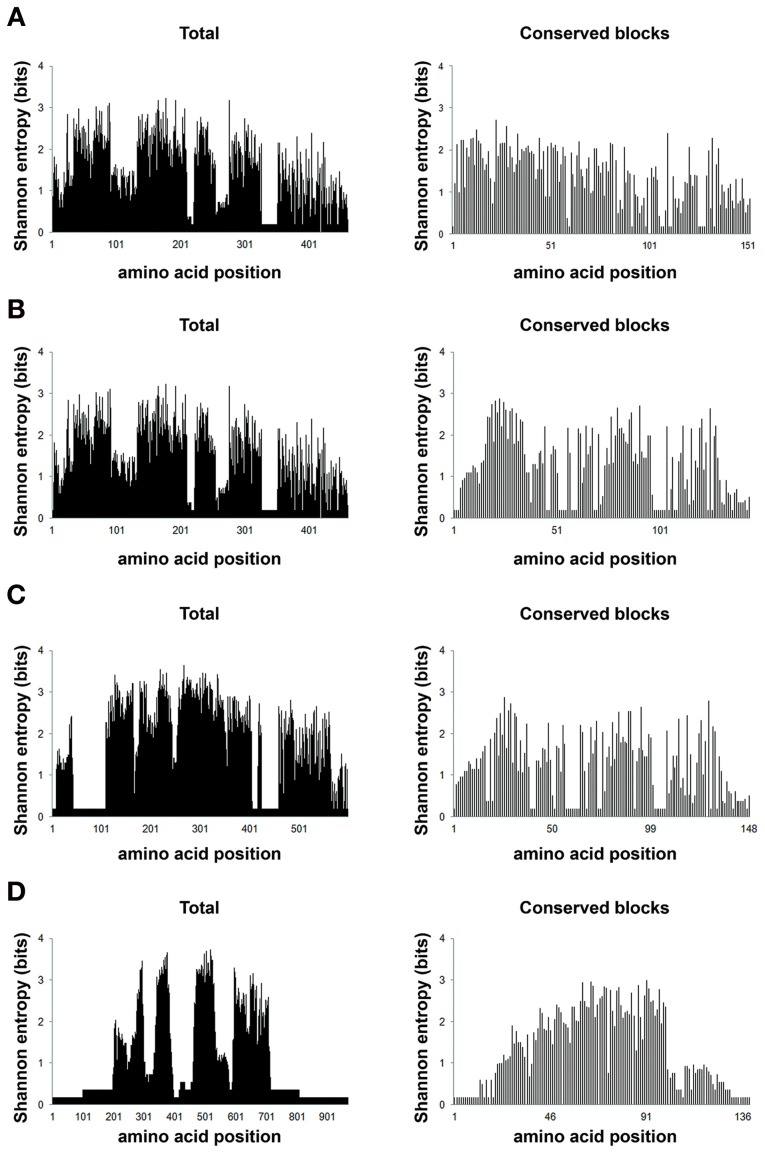
**Positional entropy.** Shannon information entropy values for the eight different amino acid alignments (full sequences and conserved amino acid blocks) were plotted according to the values generated from BioEdit (Hall, 1999). The chart **(A)** (420), is represented by alignments with 35 sequences, 460 positions (total) and 34 positions (blocks); **(B)** (150), by 34 sequences, 460 (total) and 144 (blocks) positions; **(C)** (90), 34 sequences, 598 (total) and 148 (blocks) positions; **(D)** (20) represented by alignments with 36 sequences and 967 (total) and 139 (blocks) positions. The abscissa represents the positions in each alignment and the ordinate represents the entropy values in bits for each alignment position.

Entropy analysis was used here as means to study HGT because HGT *per se* is a source of disorder in the receptor genome. Gene exchange among organisms, populations and species causes extensive genome instability, increase mutation frequency, and affects gene expression (Chia and Goldenfeld, 2011). Functional proteins (less entropic) are usually more conserved than non-functional proteins (more entropic) (Albà and Castresana, 2007) and therefore it is expected that lower entropy in conserved functional blocks as opposed to non-functional blocks. In the 4 alignments obtained with the sequences from loopback searches there are 21 different characters (20 different amino acids and gaps). The maximum entropy in this case is 4.3 bits. Thus, positions with entropies higher than 2.0 bits were considered variable, while entropies lower than 2.0 bits were considered conserved (Kawashita et al., 2009). In general, our data shows that these aligned amino acid blocks are well conserved, as indicated by the low entropy values (Figure 7).

**Figure 8:**
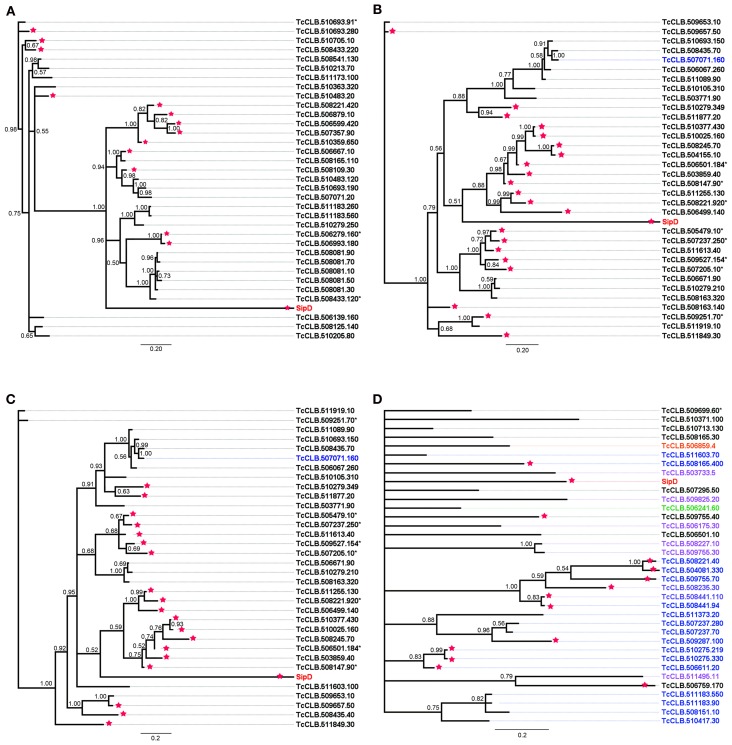
**Bayesian phylogeny of MASPs, mucins, and *Salmonella Sip*D.** Trees were inferred with the conserved amino acid blocks obtained by loopback searches. The tree's named 420 **(A)**, 150 **(B),** and 20 **(D)** were calculated from 1 × 10^7^ generations and the tree 90 **(C)** were calculated from 1.5 × 10^7^ generations. Numbers in branches represents the posterior probabilities. Letters and numbers on the right side represent GeneDB and TriTrypDB proteins access codes. Different colors indicate the types of proteins, black: MASP, blue: mucins and red: SipD (other colors, check Data Sheet 3 in Supplemental Data). Asterisks and stars within the codes represent pseudogenes and positive predictions for T3SS proteins, respectively.

To obtain a congruent analysis that could establish evolutionary relationships between *S. typhi* SipD and putative *T. cruzi* MASPs and one mucin, a larger number of amino acid sequences were obtained (Brown, 2003) by performing new searches within the *T. cruzi* genome database, using the conserved amino acid blocks from proteins 420, 150, 90, and 20 as queries. This type of approach reduces the false positives and increases the chance to find new sequences that could not be discovered by searches with the primary query. The amino acid sequences (Data Sheet 2 in Supplemental Data) and sequences obtained from database searches of different protists were aligned and submitted to Bayesian phylogenetic inferences. A total of six multiple alignments were generated (one for each *T. cruzi* proteins), comprising up to 36 sequences which included the *S. typhi* SipD, with up to 152 positions, and other 2 alignments, one comprising 179 sequences with 368 positions (different protists) and the other with 139 sequences and 444 positions (only trypanosomatids), obtained by searches in different protein databases. Apart from the phylogenetic inference obtained with the putative mucin 20, which showed a large polytomy (Figure 8D), all phylogenetic trees inferred with the MASPs (420, 150, and 90) showed the formation of a cluster comprising *S. typhi* SipD and several *T. cruzi* proteins, with posterior probabilities above 0.79 (Figure 8), suggesting a common evolutionary origin. Interestingly, a common feature of trees obtained from the alignments 420, 150, and 90 is that some putative family members of MASPs were closer to SipD than other members within the same family, indicating the presence of different groups of MASPs with distinct phylogenetic distances in relation to SipD. The sequences of putative MASPs of the inference 420 (TcCLB.510693.91 and TcCLB.510693.280) for example, were more divergent in comparison to the rest of MASPs family and forms an outgroup (Figure 8A). SipD, although more divergent than all the others proteins in the alignments, did not cluster as outgroup. The MASP (TcCLB.510693.190) that clustered with SipD (Figure 8A) was recently described by dos Santos et al. (2012) as MASP16 being highly expressed in bloodstream trypomastigote and myoblast cells. Therefore, MASP16 well as other MASPs may be involved in the invasion mechanism and calcium mobilization of *T. cruzi*, suggesting a possible homology and analogy of these MASPs with SipD.

**Figure 9:**
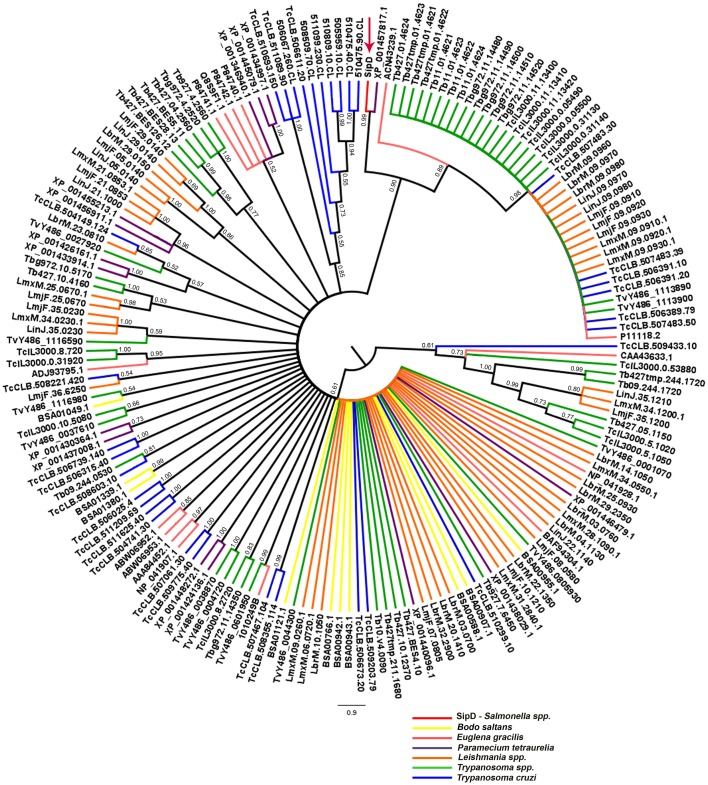
**Bayesian phylogeny with different protists and SipD.** Trees were inferred with the conserved amino acid blocks obtained by BLASTP of different protists and were calculated from 3 × 10^7^ generations. Trees are depicted as midpoint rooted. Branches colored according to genus of protists and numbers in branches represent the posterior probabilities of nodes. Letters and numbers along the branches represent GeneDB, TriTrypDB, and NCBI access codes. Arrow indicates the position of the *Salmonella Sip*D.

**Figure 10:**
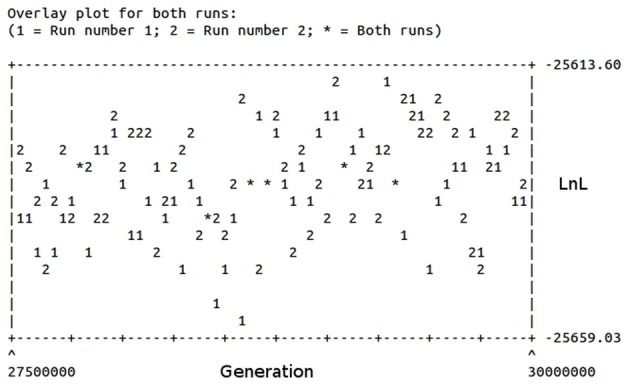
**Burn-in plot of the Bayesian inference of different protists and SipD.** The abscissa represents the generations in the search and the ordinate the LnL scores of trees. Two runs are depicted.

The phylogeny inferred using amino acid sequences of different protists was used to test if earlier branching organisms such as *Euglena gracilis*, *Paramecium tetraurelia*, and *Bodo saltans* would cluster together with SipD (Figure 9). A SipD clade with posterior probability 0.90 comprises one *Paramecium* sequence, one *Euglena* sequence and a polytomus subclade including several trypanosomatids. For this analysis the Bayesian inference was used to obtain several phylogenies in two runs with convergent LnL scores after the burn-in, around 3 × 10^7^ generations (Figure 10). The resulting phylogeny is the MrBayes “sumt” consensus of trees with converging maximum LnL scores.

**Figure 11:**
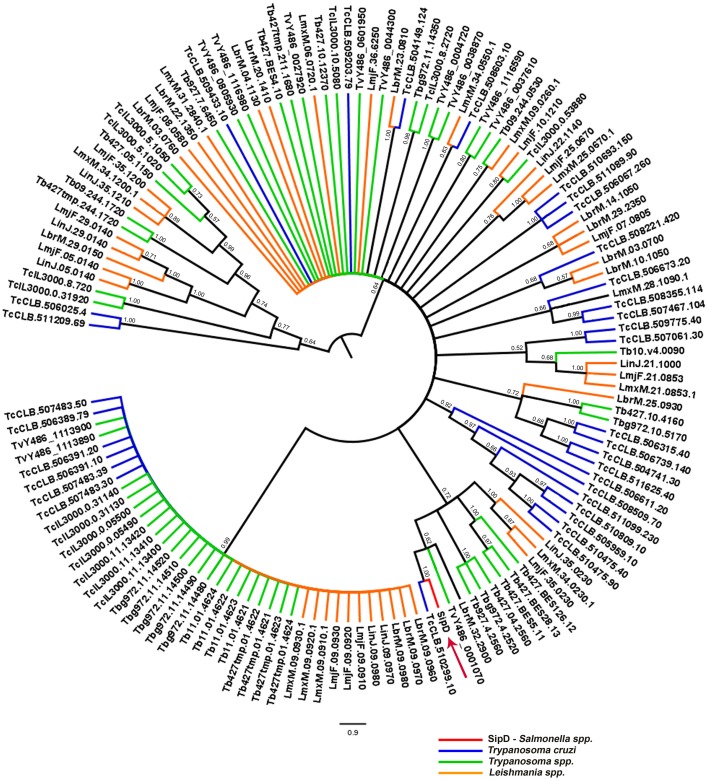
**Bayesian phylogeny of trypanosomatids and SipD.** Trees were inferred with the conserved amino acid blocks obtained by BLASTP of trypanosmatids and were calculated from 2 × 10^7^ generations. Trees are depicted as midpoint rooted. Branches colored according to genera and numbers in branches represent the posterior probabilities of nodes. Letters and numbers along the branches represent GeneDB and TriTrypDB proteins access codes. Arrow indicates the position of the *Salmonella Sip*D.

To resolve the polytomy observed in the Bayesian tree in Figure 9 a phylogeny including only amino acid sequences of trypanosomatids was inferred (Figure 11). It was observed that SipD is closer to *T. cruzi* with posterior probability 1.00 (Figure 11). This result supports our hypothesis of HGT from intracellular bacteria, more specifically from *Salmonella* spp to *T. cruzi*, because even with a large number of sequences from different trypanosomatids, SipD still clustered with *T. cruzi* sequences.

The accuracy with which phylogenies can be reconstructed, and by which HGTs can be detected, depends on the degree of divergence (Gogarten et al., 2002; Brown, 2003) and for highly divergent sequences, the number of amino acid substitutions may be saturated, resulting in loss of phylogenetic signal (Gogarten et al., 2002; Philippe and Douady, 2003; Mayrose et al., 2004). Of note, recently it has been shown that *L. tarentolae* expressing two different proteins of the MASP family trigger intracellular calcium transients in HeLa cells, presumably by injury to the cell membrane (Choi et al., 2012). This observation is consistent with our prediction of functional analogy with *Salmonella* SipD and the HGT here proposed.

## Conclusions

Our results are consistent with the hypothesis that genes involved in host cell invasion were horizontally transferred from *S. typhi* to *T. cruzi* in early evolutionary history of *T. cruzi*. Because of the marginal sequence similarities involved and long divergence dates, our data cannot rule out extreme convergent evolution. Nevertheless, the acquisition of ancestral T3SS from *Salmonella* might have contributed to the pathogenicity and singular invasion mechanisms among trypanosomatids that allowed it to actively invade host cells.

### Conflict of interest statement

The authors declare that the research was conducted in the absence of any commercial or financial relationships that could be construed as a potential conflict of interest.
